# Short Notes on the Examination of the Teeth for Bacteria, Etc.

**Published:** 1888-10

**Authors:** Vida. A. Latham

**Affiliations:** (Lond.) Ann Arbor


### Short Notes on the Examination of the Teeth for Bacteria, Etc.

VIDA. A. LATHAM, B.SC., F.R.M.S., (LOND.) ANN ARBOR.

These notes do not make any pretence at being novel or new, but I think they may be of a little assistance to dental practitioners who may be interested in the subject. We are all well acquainted with the writings of Prof. W. B. Miller, of Berlin, and Dr. G. V. Black, in the various journals, but in none do we find any allusion to the practical method. This I think is unfortunate as we, many of us, would like to have specimens to illustrate not only the normal histology and anatomy, but the pathology of the teeth in our cabinets. If those readers who may be interested in this study will kindly give their notes and methods, much valuable help will be derived from them, to the workers in this subject.

In conducting bacteriological researches, the importance of absolute cleanliness cannot be too strongly insisted upon. All slides, instruments, glass vessels and cover-glasses should be thoroughly cleansed before use. A wide-mouthed glass jar should always be close at hand, containing refuse alcohol for the reception of rejected slide preparations, or dirty cover-glasses. When required again for use, slides can be easily wiped clean with a soft rag. Cover-glasses require further treatment, for unless they are perfectly clean it is difficult to avoid the presence of air-bubbles when mounting specimens. They should be left in strong acid (hydrochloric, sulphuric, or nitric) for some hours; then washed, first with water, then with alcohol, and carefully wiped with a soft rag.

Teeth and osseous structures must be placed in a decalcifying solution, as Kleinenberg's which is made as follows:

| | | |
|---|---|---|
| ℞. | Saturated watery solution of picric acid | 100 parts |
| | Strong sulphuric acid | 2 parts |
| | Filter and add distilled water | 300 parts |

When sufficiently softened they are allowed to soak in water, to wash out the picric acid, and then transferred through weak spirit or absolute alcohol. I find Prof. Ebner's solution which is made by taking

| | | |
|---|---|---|
| Hydrochloric acid | 5 | parts, |
| Alcohol | 100 | " |
| Distilled water | 20 | " |
| Chloride of sodium | 5 | " |

gives excellent results, especially when the structures to be decalcified are placed in fresh solution from time to time. The sections may be embedded in celloidin or paraffin dissolved in chloroform. They are first placed in a mixture of ether and alcohol, after the hardening is completed, for an hour or more—then transferred to a solution of celloidin in equal parts of ether and alcohol, and left there usually for several hours. The corks ready cut for the clamps of the microtome are smeared over with a solution of celloidin and applied with a glass rod to the surface which is to receive the object. Let the corks be placed aside for the film of celloidin to harden. The specimen should be allowed to stay in the celloidin solution for from one to twenty-four hours and when ready removed with forceps and placed on the prepared cork. A little of the solution which is of syrupy consistence, is allowed to fall on a piece of tissue to cover it completely, and finally place the mounted specimen in 60–80 per cent. of alcohol to harden the celloidin. The specimen may be cut the next day. This is applicable for all tissues. The celloidin in the section disappears in the process of clearing with clove oil. In using this imbedding agent, flood the knife constantly with alcohol and transfer the sections from the blade with a camel's hair brush and place in alcohol. If cut with the freezing microtome and having been hardened in spirit they must be *well* soaked in water before freezing—if hardened in Muller's fluid it can be done at once.

METHOD OF STAINING.

Cocci stain well with watery solutions of gentian-violet, methyl-violet, fuchsin, methylene blue, and bismarck brown.

For a zoogloea or pellicle of micrococci, transfer bodily to a watch-glass containing the dye, leaving it there until deeply tinted, then taking it out with a needle, washing in water, and then in alcohol till the excess of color is removed. Then transfer to a glass slip, spread well out, and a drop of clove oil placed on it; after a minute or two the clove oil is drained off, add a drop of Canada balsam (and I prefer the preparation made by the Palmer Slide Co.) and cover.

Cocci in the tissues of the teeth, etc., may be stained by immersing the sections in an aqueous solution of gentian violet or in aniline-gentian-violet solution, then rinse in water, decolorize in alcohol, treat with clove-oil, and preserve in balsam; or after washing with alcohol they may be rinsed with water and stained for half an hour with Weigert's picro-carmine; wash again successively in water, alcohol, clove-oil and transfer with a copper lifter to a clean glass slide. Dry the preparation by pressure with a piece of filter paper folded four times, and preserve in Canada balsam dissolved in xylol or benzole. Gram's method is very useful and gives satisfactory results—using as an *after*-stain rosin in the following manner: after decolorisation place in a weak alcoholic solution of rosin (2 or 3 drops of a concentrated alcoholic solution added to a watch-glassful of alcohol) till stained a delicate pink. Then rinse in fresh alcohol, treat with clove oil, and mount in balsam. Many other stains may be experimented with and probably with success, but the above are the only ones I have as yet tried in dental tissues.

MICROCOCCUS FŒTIDUS, ROSENBACH.—Small oral cocci—they are best cultivated in agar—agar and develop gas bubbles and a fœtid odor. Isolated from carious teeth. Possibly closely allied to, if not identical with micro crepusculum of Cohn.

STREPTOCOCCUS.—Two species of streptococci are believed to be intimately connected with caries of the teeth. The first occurs in the form of cocci, diplococci, and chains, which develop very rapidly in nutrient gelatine, speedily converting it into a turbid liquid. They are agents of lactic acid fermentations.

The second occur as very small cocci, rarely in chains, which rapidly liquefy nutrient gelatine. There are also associated with these two species of micrococci and a spirillum (sp. sputigenum).

SPIRILLUM SPUTIGENUM, LEWIS—Presents curved rods very similar to the comma-bacilli of Koch. I believe many have failed with repeated attempts to cultivate these baccili, and, therefore, maintain that they are quite distinct biologically from the spirilla associated with Asiatic cholera. They may be cultivated in an acid nutrient gelatine, and occur with other bacteria in saliva, and in scrapings from carious teeth. In caries the advancing margin of the diseased area of the tubules of the dentine appear to be widened (Klebs, Leber, and Rottenstein) and surrounded by bright rings. Presently the tubules are seen to contain a granular mass, which turns blue when treated with iodine and increases in bulk at the expense of the bright rings. This mass will be found to consist of baccilli and micrococci, and according to Klebs it is they that are the destroyers of the dental tissue, as they possess the power of decalcifying it. They are enabled to penetrate the substance of the teeth by the accidental formation of cracks or flaws in the enamel.

The tartar also contains multitudes of bacilli and micrococci mingled with calcareous particles. He maintains that the organisms are able to precipitate calcium salts from the nutrient materials they assimilate. A very common result of caries is inflammation of the pulp or of the alveolar periosteum. The irritants which directly induce it are perhaps the bacteria which are present in the disintegrated dental substance, and set up septic decomposition around them. The inflammation of the pulp and periosteum may pass into suppuration. In this case the surface of the gum in the neighborhood of the diseased tooth is red and swollen (gum-boil or parulis), and presently suppuration sets in, extending to the deeper tissue of the gum, forming an alveolar abscess. This breaks externally and if the suppurative process around the root of the tooth continues, we may have formed an alveolar sinus or fistula.

Sometimes the inflammation extends beyond the region of the root and gives rise to an extensive periostitis of the jaw, large abscesses may form, and necrosis of the maxillæ may result. The microbe which is most common in decayed teeth is very polymorphic. Micrococcus, bacterium, chains and filaments, are only different phases of the same plant, which also produces lactic fermentation in the mouth, and formation of lactic acid. Miller has succeeded in producing this disease in sound teeth artificially, and according to his experiments the best dentifrice for the destruction of microbes is a solution of corrosive sublimate (mercuric chloride), one part in 1,000, which can be further diluted by four parts of pure water. I use as a double stain the following process: First stain the specimen with methyl-violet, then dip for a moment in iodine solution, wash in water or weak alcohol, then steep for some minutes in picrocarminate of ammonia of which the color is made lighter by washing with absolute alcohol and clove oil, mount in balsam. The nuclei of the cells are then of a carmine red, and the bacteria violet; the rest of the preparation is of a much paler color. To isolate Nasmyth's cuticle prepare a thin layer from the crown of a tooth freshly erupted, or just previous to its eruption; grind this, taking care to leave intact the outer surface of the enamel. This is then placed between two plates of glass moistened with a drop of water upon the field of view; a drop or two of hydrochloric acid is then added, and soon a thin membrane will be seen to separate from the edge of the enamel, driven off by the bubbles of gas which issue on every side.

As yet the bacteria and bacilli researches in dental pathology are in their infancy, but we may hope to have much valuable assistance in arresting if not entirely curing these diseases at no very late day. The histologist will find that such work as the above will give him much pleasant employment for winter evenings provided good illumination and apparatus be used.

BIBLIOGRAPHY.

Trouesart, Microbes, Ferments, and Fungi.

Miller, Deutsche Med. Woch. Nr. 25 u. 36.

Miller, Cent. f. d. Med. Wiss. 13, Arch. f. Exp. Path. xiv 1882.

Klenke, Die Verderbniss der Zahne: Leipzig, 1850.

Klebs, Arch. f. Exp. Path. v.

Lewis, Med. Times and Gazette, Sept. 20, 1884.

Arndt, Beob. an Spirochaeta Denticola. Arch: f. Path. Anat. Phys. und Klin. Med. 79.

Klein, Micro-organisms.

Woodhead & Hare, Practical Mycology.
Rosenbach, Mikro-org. bie. den Wundinfectionskraukheiten. 8. 77.
Klein, Micro-organisms.
Woodhead & Hare, Practical Mycology. Edin. and Journal of the Royal Miros : Soc.
Black G. V. Formation of Poisons by Micro-Organisms.
Leber & Rottenstein, Dental Caries.
Magitot's Dental Caries.
Dental Cosmos and the various Dental Journals.

